# High-Performance Humidity Sensor Based on the Graphene Flower/Zinc Oxide Composite

**DOI:** 10.3390/nano11010242

**Published:** 2021-01-18

**Authors:** Muhammad Saqib, Shenawar Ali Khan, Hafiz Mohammad Mutee Ur Rehman, Yunsook Yang, Seongwan Kim, Muhammad Muqeet Rehman, Woo Young Kim

**Affiliations:** 1 Faculty of Applied Energy System, Major of Electronic Engineering, Jeju National University, Jeju 63243, Korea; saqibmuhammad@jejunu.ac.kr (M.S.); shenawaralikhan@jejunu.ac.kr (S.A.K.); mutee1990@jejunu.ac.kr (H.M.M.U.R.); yunsuk0001@jejunu.ac.kr (Y.Y.); pea8543@jejunu.ac.kr (S.K.); or muqeetmughal88@gmail.com (M.M.R.); 2 Department of Electronic Engineering, Jeju National University, Jeju 63243, Korea

**Keywords:** functional material, graphene flower and ZnO composite, sol–gel method, ultra-sensitive humidity sensor, fast response/recovery time, health monitoring applications

## Abstract

Performance of an electronic device relies heavily on the availability of a suitable functional material. One of the simple, easy, and cost-effective ways to obtain novel functional materials with improved properties for desired applications is to make composites of selected materials. In this work, a novel composite of transparent n-type zinc oxide (ZnO) with a wide bandgap and a unique structure of graphene in the form of a graphene flower (GrF) is synthesized and used as the functional layer of a humidity sensor. The (GrF/ZnO) composite was synthesized by a simple sol–gel method. Morphological, elemental, and structural characterizations of GrF/ZnO composite were performed by a field emission scanning electron microscope (FESEM), energy-dispersive spectroscopy (EDS), and an x-ray diffractometer (XRD), respectively, to fully understand the properties of this newly synthesized functional material. The proposed humidity sensor was tested in the relative humidity (RH) range of 15% RH% to 86% RH%. The demonstrated sensor illustrated a highly sensitive response to humidity with an average current change of 7.77 μA/RH%. Other prominent characteristics shown by this device include but were not limited to high stability, repeatable results, fast response, and quick recovery time. The proposed humidity sensor was highly sensitive to human breathing, thus making it a promising candidate for various applications related to health monitoring.

## 1. Introduction

Measurement of humidity is an essential factor for various industries including semiconductor fabs, pharmaceutical, food and beverage, agriculture, textile, refineries, paper and chemical industries, etc. [1]. The significance of humidity sensors can be estimated by evaluating its market value which was USD 901.7 million in 2018 and is expected to reach USD 1.6 billion by 2025 [2,3]. Among the available humidity sensors, relative humidity sensors are most widely used with maximum market shares [2,3]. Relative humidity sensors can be classified into several types depending on their operation such as resistive, capacitive, impedance, field-effect transistor (FET), optic-fiber, surface acoustic wave (SAW), etc. [4,5,6]. Resistive, capacitive, and impedance-based humidity sensors have similar device structure and are commonly used due to their facile fabrication, simplicity of operation, cost-effectiveness, and low driven power [5,6,7]. The performance of any type of humidity sensor is strongly dependent on the functional material which can bring good change to its electrical properties with respect to variation in humidity.

Presently, researchers are primarily focused on improving the characteristics of the functional material in humidity sensors by experimenting with various advanced materials and composites of existing materials and, therefore, several types of materials, including ceramics, polymers, biowastes, metal oxides, 2D materials, and their composites have been employed in humidity sensors [8,9,10,11,12,13,14]. Primarily, metal oxides such as ZnO, SnO_2_, Fe_2_O_3_, CuO, and TiO_2_ have been engaged in humidity sensing applications due to their unique electrochemical and electronic properties [15,16,17,18]. Among them, ZnO and its composites are widely used for humidity sensors due to their easy fabrication, low cost, high surface area, and high mobility of charge carriers [15,19,20,21,22,23,24]. Diverse structures of ZnO, including nanowires, nanorods, nanoparticles, and thin films have been used to improve various characteristics of a humidity sensor including its response time, recovery time, and range [25,26,27,28,29]. However, humidity sensors based on pure ZnO lack good sensitivity, limiting their use for various practical applications. Therefore, in order to use ZnO as the active material of a humidity sensor, it must be mixed with some other suitable material to overcome its deficiencies. Materials like metals, semiconductors, polymers, ceramics, and 2D materials have been used by different researchers to improve the humidity sensing properties of ZnO-based sensors [17,19,20,21,22,23,24].

The composite of ZnO with different 2D materials like graphene, MoS_2_, and WS_2_ illustrated great response to the change in humidity owing to their high surface to volume ratio, large surface area, and improved chemical and electrical properties [30,31,32]. Among these 2D materials, graphene was given great attention due to its excellent properties of high conductivity, the large specific surface area of 2600 m^2^ g^−1^, being optically transparent and having high chemical stability. Therefore, graphene and its derivative composites were widely investigated for the application of sensing change in humidity like SnO_2_/reduced graphene oxide (RGO) [33], methyl red/graphene flakes [34], black phosphorous (BP)/graphene [35], lignosulfonate/RGO [36], BP/graphene oxide (GO) [37], non-woven fabric/GO [38], orange die/graphene [39], graphene/polyvinyl alcohol (PVA)/SiO_2_ [40], chitosan/graphene quantum dots [41], etc. Studies have shown that the structure of graphene has a huge impact on the properties of the humidity sensor; therefore, graphene has been used in different forms with ZnO to enhance the humidity sensing behavior of ZnO. These different structures of graphene and its derivatives include graphene flakes/ZnO [30], graphene foam/ZnO [42], graphene quantum dot/ZnO nanowires [43], ZnO/ polyvinylpyrrolidone (PVP)-RGO [44], ZnO/GO [45], etc. Improvement is still required in developing a long-range, stable, more sensitive, fast response and quick recovery time ZnO-based humidity sensor that is easy to fabricate. 

Herein, we propose a simple approach to fabricate and characterize the humidity sensor based on the composite of ZnO with graphene in the form of graphene flowers, a type of graphene that is directly synthesized based on a high rate CVD method using a bottom-up system. The proposed sensor showed a swift response time of a few milliseconds and a small recovery time of only 3 s, excellent stability, and sensitivity to long-range relative humidity (RH)%. ZnO thin film was grown by the sol–gel method, and the thin film of graphene flowers was spray coated on ZnO. GrF/ZnO composite was characterized by a field emission scanning electron microscope (FESEM), energy-dispersive spectroscopy (EDS), and an X-ray diffractometer (XRD). The prepared sensor’s electrical response was measured by an LCR meter and a precision source measurement unit at different RH% levels. 

## 2. Experiment

### 2.1. Materials

For the preparation of graphene flower (GF)–ZnO composite, zinc–acetate–dihydrate (ZAD), monoethanolamine (MEA), ethanol isopropyl–alcohol (IPA), acetone, and deionized water were purchased from Sigma-Aldrich, Seoul, Korea. Graphene flower (GF) solution in methyl-ethyl-ketone (MEK) (99.9 wt%) was purchased from InALA, Kobe, Japan. All materials were used without modification while glass substrate and silver paste for patterning electrodes were purchased from MTI Korea.

Nine types of different salts LiCl, CH_3_COOK, CaCl_2_, K_2_CO_3_, NaBr, CuCl_2_, NaCl, KCL, and K_2_SO_4_ were purchased from DAEJUNG Materials Korea to make supersaturated solutions for achieving different RH% levels. 

### 2.2. Device Fabrication

**Figure 1:**
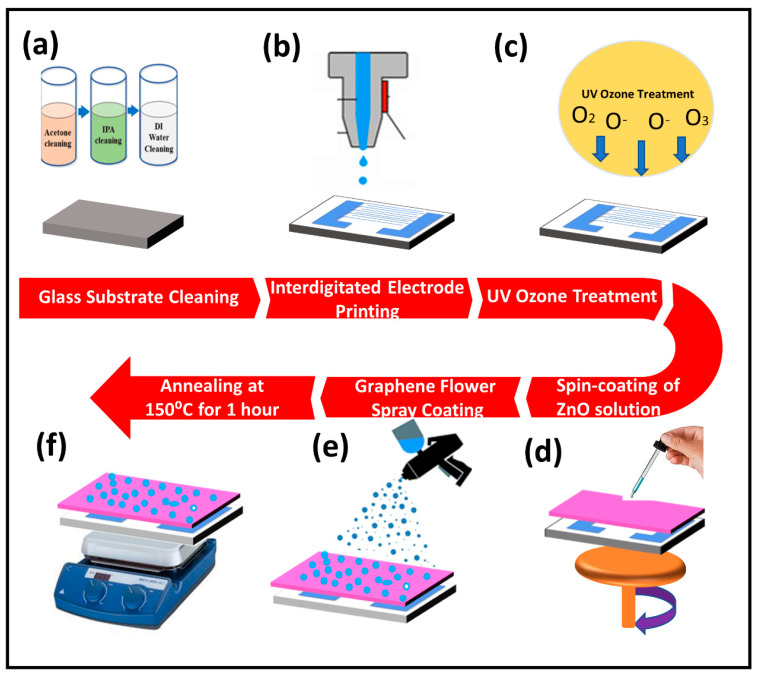
Process flow diagram: (**a**) substrate cleaning (**b**) electrode printing (**c**) UV-ozone treatment (**d**) sol–gel ZnO thin film deposition (**e**) graphene flower (GrF) spray coating (**f**) annealing for 1 h.

The entire process of device fabrication is illustrated in Figure 1. The glass was used as a substrate after cleaning it with acetone, isopropanol, and DI water for 30 min each followed by bath sonication. Interdigitated electrodes (IDE’s) were fabricated on this cleaned glass substrate by using the technique of screen printing. The sample was treated with UV-ozone before depositing a thin film of active layer in order to enhance its adhesion to the surface. For the preparation of ZnO thin film, an optimum study for the best molar solution of ZAD was performed by using three different molar solutions 1 M, 0.5 M, and 0.1 M of ZAD in ethanol, and it was found that the 0.5-M solution was the best choice based on the obtained results as 0.1-M solution required a higher number of spin coating cycles to obtain the desired thickness while achieving a thin-film with the 1-M solution was not possible due to its high density. After UV-ozone cleaning of IDE’s, a 0.5-M solution of ZAD and MEA in ethanol was spin coated on the substrate in two steps (500 rpm for 30 s and 1500 rpm for 15 s) followed by sintering at 150 °C for 20 min and the same process was repeated seven times to obtain a uniform thin film followed by annealing for one hour [46,47]. A solution of GrF in MEK solvent was spray coated on the already spin-coated thin film of ZnO followed by heating the sample at 150 °C for 1 h on a hotplate.

### 2.3. Device Characterization and Measurements

Morphology of ZnO thin film and GrF/ZnO composite was investigated by a field emission scanning electron microscope (FESEM) (TESCAN, Brno, Czech Republic, MIRA3) while the gap between the deposited electrodes was visualized with the help of an optical microscope (Olympus BX60, Tokyo, Japan). Figure S1 (supplementary data) shows the microscopic image of as-deposited electrodes with its finger width and distance between two fingers along with the complete picture of whole interdigitated electrodes. Energy-dispersive spectroscopy (EDS, Brno, Czech Republic) was used for the elemental analysis of the prepared composite films. Phase analysis of ZnO and GrF/ZnO composite thin film was performed by an X-ray diffractometer (Empyrean) using Cu target at the wavelength of 1.5406 Å, step size 0.02° and scanning range from 5° to 80°.

**Figure 2:**
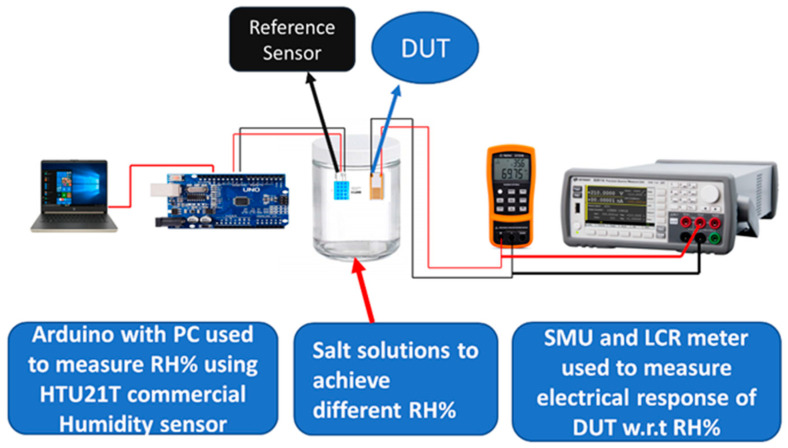
Experimental setup used for electrical measurement of the device under test (DUT).

The experimental setup is shown in Figure 2; a supersaturated solution of different salts was used to achieve various RH% levels in airtight glass jars to examine sensor response to humidity. The value of RH% achieved in these jars is given in Table 1. In the literature [9,36], the reported RH% level of different salt super-saturated solutions are lithium chloride (11%), potassium acetate (23%), calcium chloride (33%), potassium carbonate (43%), sodium bromide (57%), sodium chloride (67%), copper chloride (87%) and potassium sulfate (97%), but we achieved minimum RH% of 15% with LiCl, CH_3_COOK(23%), CaCl_2_ (32%), K_2_CO_3_ (46%), NaBr (60%), CuCl_2_ (67%), NaCl (72%), KCl (80%) and maximum RH% of 86% from K_2_SO_4_ super-saturated solution with an accuracy of ±2%. A commercially available humidity sensor, HTU21D, was used along with an Arduino board to calibrate the humidity level of supersaturated solutions. The device under test (DUT), i.e., the fabricated sensor, was placed in the desired RH% environment by placing it inside the jar of corresponding salt solution for electrical measurements with respect to humidity. The used experimental setup is shown in Figure S2.

Change in the electrical properties of proposed humidity sensor with respect to change in RH% were measured with different electronic devices such as LCR meter (KEYSIGHT U1733C) which was used to measure impedance and capacitive response of sensors with respect to different RH% levels. A precision source measurement unit (KEYSIGHT 2911A) was used to measure current change with respect to RH%, response time, recovery time, stability, and different application tests of the proposed humidity sensor.

## 3. Results and Discussion

### 3.1. Characterization of ZnO and GrF/ZnO Composite 

An FESEM was used to examine the morphology of GrF, ZnO and GrF/ZnO thin films. For the uniform thin film of ZnO deposited by the sol–gel method, multiple spin coatings were required [47,48]. Therefore, a detailed study was done to find the effect of different spin coatings on ZnO thin films and samples with 1, 3, 5, 7, 9 and 11 cycles of spin-coating were examined by using FESEM and EDS analysis whose results are shown in Figure S3. The obtained results demonstrated that the thin film achieved after seven cycles of spin coating gave the best results and provided a uniform thin film, whereas the thin film of ZnO obtained for spin coating cycles of less than seven was non-uniform with different thicknesses at the center and edges.

**Figure 3:**
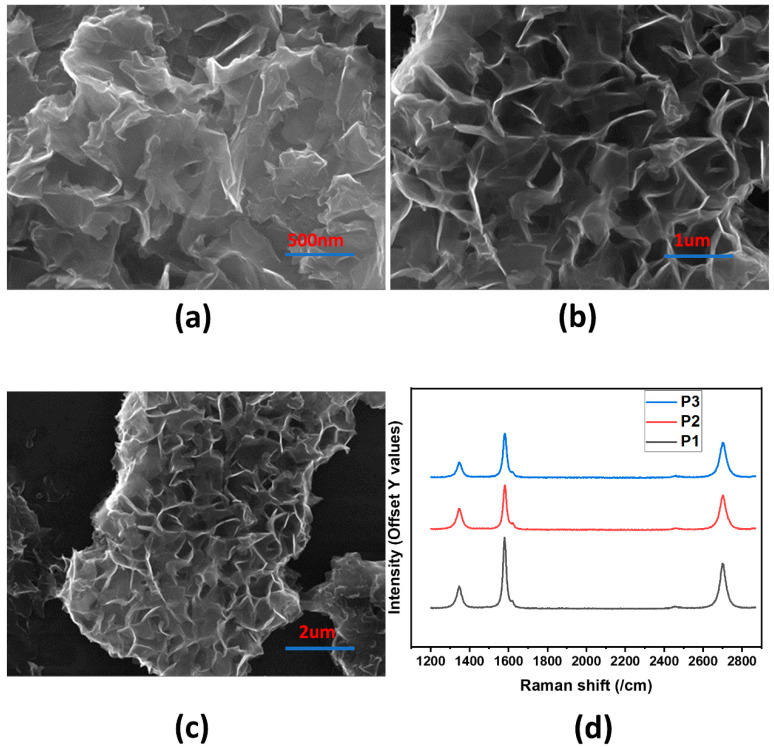
Characterization of graphene flowers, (**a**–**c**) field emission scanning electron microscope (FESEM) analysis, (**d**) Raman analysis.

Figure 3a–c, illustrates the FESEM images of GrF where the bright regions represent the edges of GrF; thus, a flower-like formation can be seen. FESEM images clearly show the high surface area and 3D structure of GrF. Figure 3d depicts the Raman spectroscopy analysis of graphene flowers at three different points. At all three points P1, P2 and P3 the Raman shift is approximately identical. From the ratio of G and 2D peaks, we can deduce that GrF has multiple layers.

**Figure 4:**
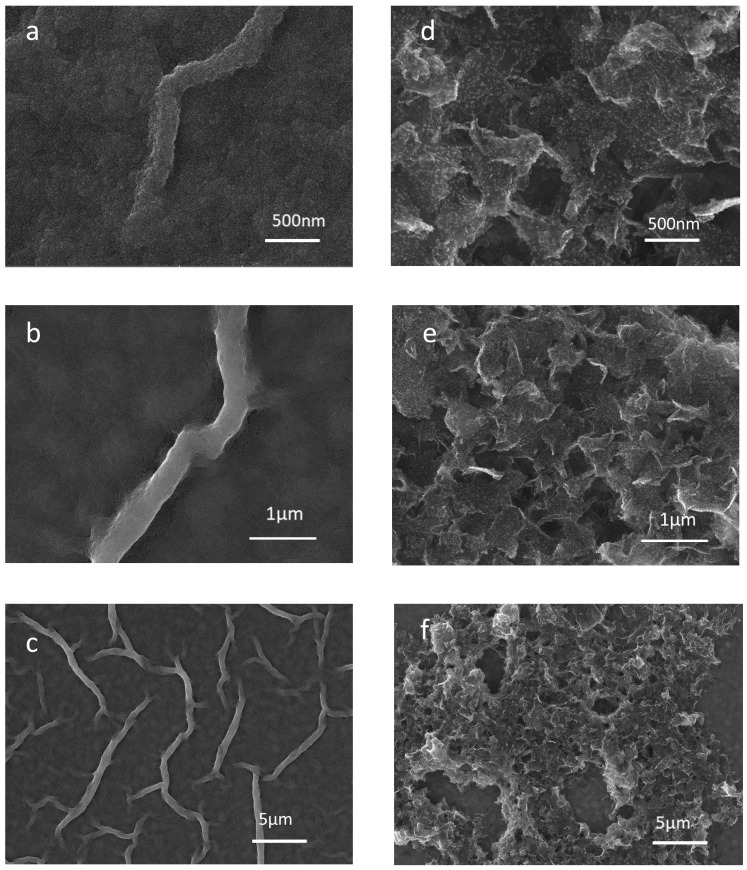
FESEM analysis of grown thin films, (**a**–**c**) SEM images of ZnO thin film at different magnifications; (**d**–**f**) GrF/ZnO composite SEM images at various magnifications.

Figure 4a–c illustrate the surface morphology of the optimized ZnO thin film, revealing that ZnO has a relatively smooth surface. Figure 4d–f shows the morphology of the GrF/ZnO composite, which clearly shows that it has pores and a greater surface area compared to pristine ZnO thin film. FESEM results confirmed that the GrF/ZnO composite has a high surface area to volume ratio and pores that are essential for the functional material of any humidity sensor. 

**Figure 5:**
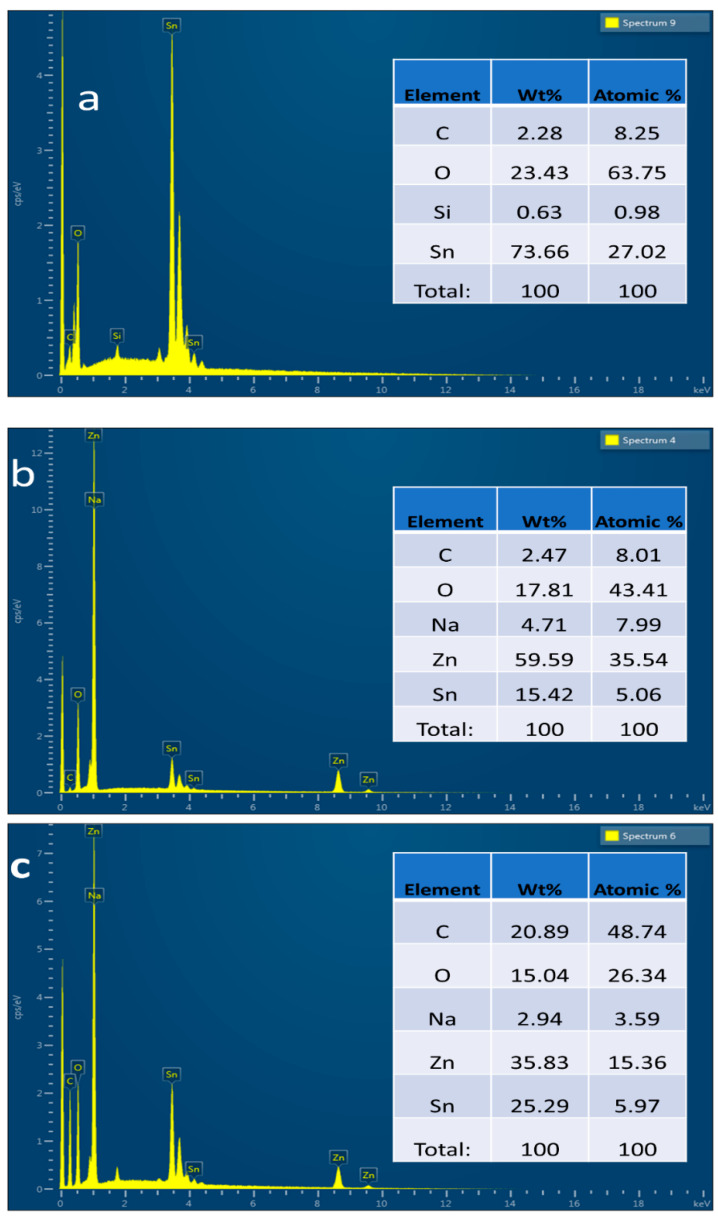
EDS analysis of, (**a**) EDS spectrum of fluorine tin oxide (FTO) glass (that was used as a substrate); (**b**) EDS spectrum of ZnO thin film; (**c**) GrF/ZnO composite.

EDS analysis is usually used to confirm the elemental composition of the given samples; therefore, EDS analysis was performed to verify the successful synthesis of ZnO and GrF/ZnO thin films. Samples were made on fluorine tin oxide (FTO)-coated glass to perform EDS analysis. EDS of FTO glass, ZnO thin film, and GrF/ZnO is shown in Figure 5a–c. It is clearly shown in the EDS spectrum of FTO glass that tin (Sn) and oxygen were the only dominant elements with wt% of 73.66% and 23.43%, respectively. Figure 5b illustrates the EDS spectrum of ZnO thin film deposited on FTO glass by the sol–gel method; it confirms that ZnO thin film is grown successfully as zinc (Zn) was present in the spectrum with wt% of approximately 60%. Synthesis of GrF/ZnO was also confirmed by EDS analysis shown in Figure 5c, illustrating the presence of carbon (C), zinc (Zn) oxygen (O), and tin (Sn) at the wt% of 21%, 15%, 36%, and 25%, respectively.

**Figure 6:**
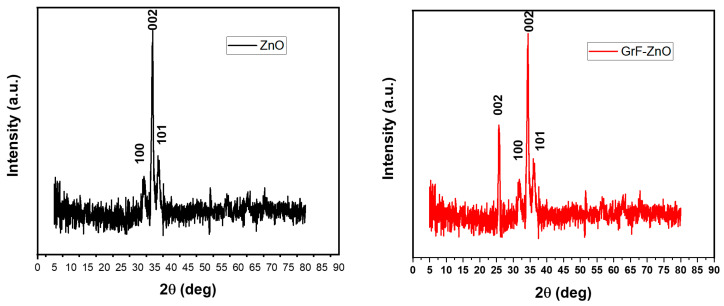
XRD analysis of (**a**) ZnO thin film; (**b**) GrF/ZnO composite.

XRD analysis was performed to investigate the crystal structure of prepared ZnO and GrF/ZnO thin films using the X-ray diffractometer (Empyrean) at a step size of 0.02° from 5° to 80°. XRD analysis of ZnO thin film exhibited the peaks at 31.7°, 34.4°, and 36.4° as shown in Figure 6a, corresponding to lattice planes (100), (002) and (101), respectively, that confirm the hexagonal wurtzite structure of ZnO thin film according to joint committee of powder diffraction standards (JCPDS) card no. 36-1451 and also according to the literature [48]. In Figure 6b, XRD analysis of GrF/ZnO composite is presented that shows the extra peak at 2θ = 26°, which corresponds to (002) lattice plane of graphene [49,50] and peaks at 31.7°, 34.4°, and 36.4° correspond to lattice planes (100), (002) and (101) of ZnO, respectively. 

### 3.2. Electrical Response of GrF/ZnO Composite with Respect to RH%

**Figure 7:**
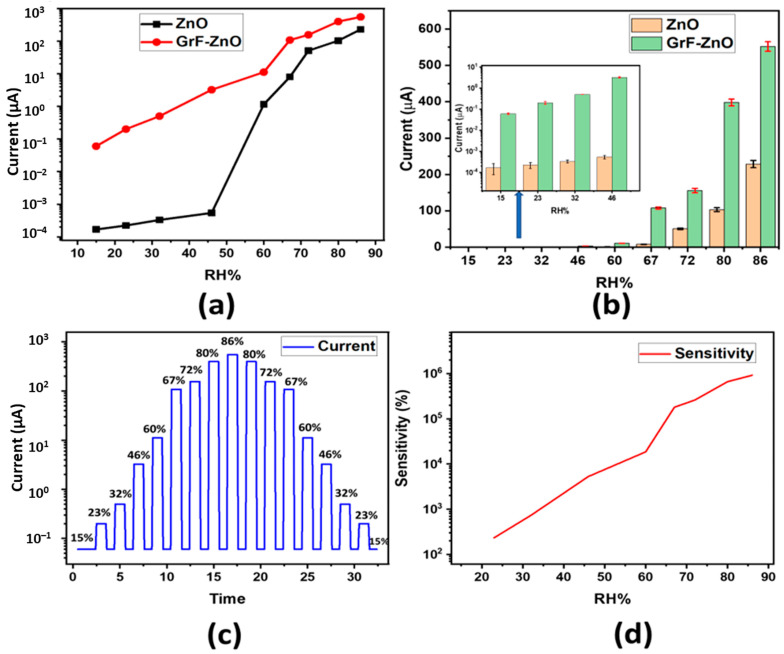
Electrical response with respect to RH%, (**a**) current vs. RH%; (**b**) repeatability analysis and comparison of current at different RH% (inset shows the current comparison for low humidity levels); (**c**) current response of the GrF–ZnO composite-based sensor under switching RH; (**d**) sensitivity of GrF–ZnO sensor.

The experimental setup shown in Figure 2 was used to measure the proposed sensor’s electrical response with respect to RH%. Different salt solutions were used to achieve various RH% levels, ranging from 15% to 86% RH% by using supersaturated solutions of LiCl and K_2_SO_4_, respectively. A biased voltage of 3 V was given to the fabricated sensors using the KEYSIGHT B2911A Precision Source/Measure Unit, and the current was measured by placing the sensor at different RH% levels. Figure 7a shows the change in current magnitude with respect to RH%. GrF/ZnO composite-based humidity sensor exhibited considerable change in the value of current at different RH% levels ranging from a few nanoamperes to hundreds of microamperes. In the proposed sensor, we utilize graphene flowers (a 3D structure with large surface area) to make composite with ZnO for sensing humidity response. These graphene flowers are not electrically connected with each other. Initially, the active layer comprising ZnO and graphene flowers had a high resistance; however, resistance of this active layer can be decreased by forming an electrical connection between GrF such as through the absorption of water vapors. When humidity increases, water vapors fall on the sensing film, which makes an electrical connection between GrFs, resulting in an overall decrease in the value of resistance hence, allowing more current to flow between the two sets of IDEs. The 3D structure of GrF plays a vital role in providing a large surface area for the absorption of water vapors that results in a considerable increase in the conductivity of the ZnO/GrF active layer. The trend of change in conductivity shown by our proposed device with absorption of water vapors is in line with other graphene-based humidity sensors reported in the literature [4,29,36,41]. Smith et al. [4] reported a decrease in resistance of graphene sheet with an increase in humidity level. S. Ali et al. [33] showed that resistance of graphene flakes/methyl red composite was decreased as RH% increases. Moreover, as shown in Figure 7a, a pristine ZnO-based device showed random current change over the RH% change; initially, the change is approximately zero, whereas, after 60% RH, it shows a sharp change. It can be seen in Figure 7b that the GrF/ZnO-based humidity sensor has shown a better current change over the whole RH% range as compared to the pristine ZnO-based humidity sensor. The characteristic of repeatability is also shown in Figure 7b, which exhibits the average measured current versus relative humidity for both ZnO and GrF/ZnO-based humidity sensors. The error bars indicate the standard deviation of the data from their average value, with a maximum standard deviation of 10 μA and 13 μA for ZnO and GrF-ZnO-based humidity sensors, respectively. These data represent the repetition of the experiment nine times. Figure 7c illustrates the current response for the GrF/ZnO-based sensor upon exposure to different RH% levels. The reversibility and reproducibility of the sensor was examined through exposure/recovery cycles for the sensor exposed from low RH% to high RH%, and then conversely from high RH% to low RH%. Figure 7d shows the sensitivity graph of the GrF/ZnO-based humidity sensor. Sensitivity of the proposed sensor was found using the Equation (1).

(1) $$S=\frac{I_{RH}-I_{RHo}}{I_{RHo}}*100\%$$

where *I_RHo_* is the value of current at 15% RH and I_RH_ is the value of current at exposure RH%.

Similarly, as current change over RH% was measured, impedance and capacitance change with respect to RH% was also measured using the KEYSIGHT U1733C LCR meter (V_o_ = 0.7 V at different test frequencies of 1 kHz, 10 kHz and 100 kHz). Figure S4 shows the capacitance response with respect to RH% for the GrF/ZnO-based humidity sensor. It shows that with the increase in the level of RH%, the value of capacitance also increases whereas impedance decreases as RH% increases as shown in Figure S5. The inverse relation between RH%, and impedance can also be verified from the previous current vs. RH% result and the literature [4,36,41]. 

**Figure 8:**
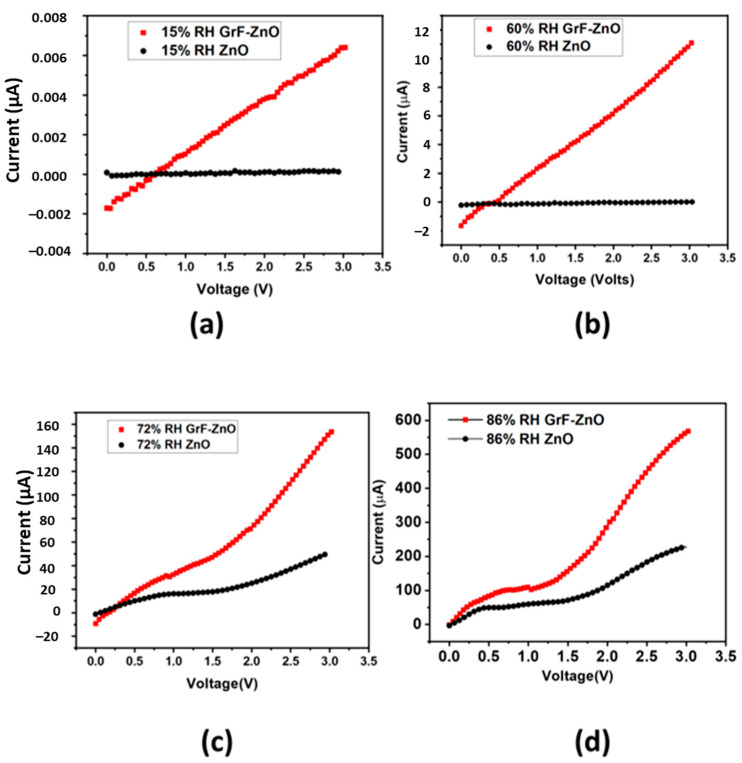
Current vs. voltage at different RH%, (**a**) 15% RH; (**b**) 60% RH, (**c**) 72% RH and (**d**) 86% RH.

Figure 8 depicts the current over voltage response of both electronic devices i.e., pristine ZnO and GrF/ZnO-based humidity sensors placed at a particular RH% level. Devices were placed in a desired RH% level jar initially for 20 min to stabilize the closed jar’s humidity followed by applying the voltage sweep from 0 V to 3 V using the KEYSIGHT B2911A Precision Source/Measure Unit (interfaced with a PC), and the current was measured correspondingly. These electrical results shown in Figure 8a–d confirmed the ohmic behavior of the proposed device and also verified the previously obtained results that showed a direct relation between increase in current and increasing levels of humidity. Figure 8a–d clearly shows that the GrF/ZnO-based humidity sensor established a significant current change with respect to the applied voltage sweep at each RH% level that confirms its ohmic behavior and the possibility of operation at low voltages. Figure 8a,b shows that pristine the ZnO-based humidity sensor has approximately zero current change with respect to applied voltage sweep at low humidity levels and low current change at high RH% levels compared to the GrF/ZnO-based sensor whose results are provided in Figure 8c,d. The obtained electrical results present a strong case for the GrF/ZnO-based humidity sensor with respect to operational power as well as it requires less power as compared to the pristine ZnO-based sensor.

**Figure 9:**
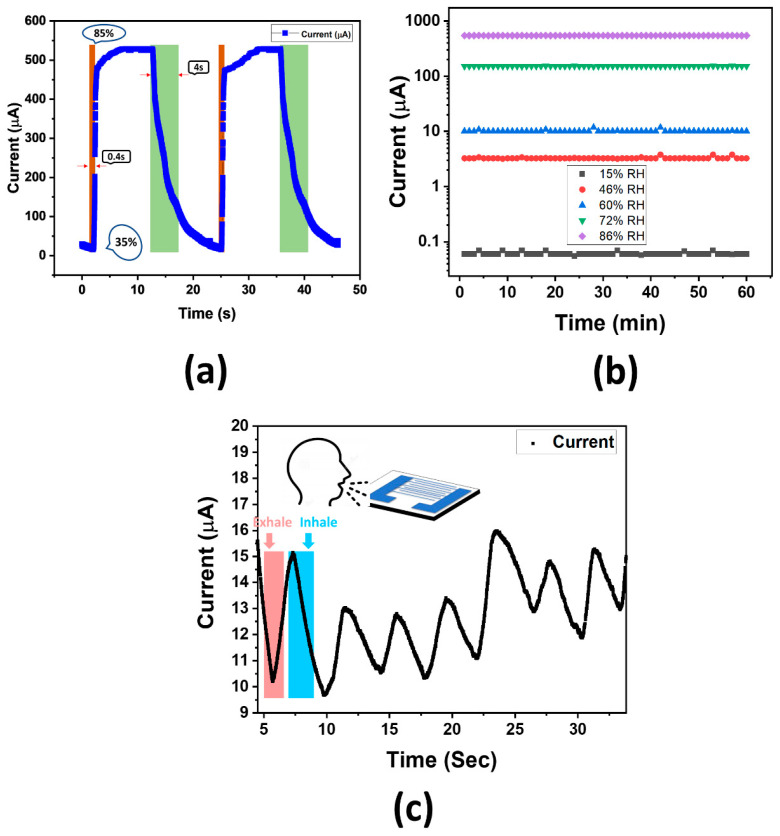
(**a**) Response and recovery time; (**b**) stability test of the proposed sensor, (**c**) sensor response to normal human breathing.

Response and recovery time are the essential features for any humidity sensor, and these are the main characteristics based on which sensors’ performance can be evaluated. The response time is defined as the time required for the humidity sensor to reach 90% of the current change (ΔI) when the sensor is exposed to a given level of humidity. The recovery time is defined as the time needed to recover to 90% of the initial baseline after turning-off the humidity. Response and recovery time of the proposed sensor were measured by changing its position from one jar (low-level RH%) to the other jar (high-level RH%) which takes few minutes; therefore, response and recovery time measurement using this setup was not possible, so an alternate method was used. For measuring the response time, proposed sensor was moved from open air 35% RH to 85% RH jar and recovery time was measured by moving back the proposed sensor from 85% RH jar to open air. Figure 9a shows the response and recovery time graphs of our proposed sensor, illustrating a fast response (0.4 s) to humid air along with a short recovery time of just 4 s. We compared our results with the earlier reported humidity sensors based on graphene sheet and found that these results are better than its counterparts [4,29,42]. Here we would like to mention that earlier we tested our humidity sensor in the range of 15% to 86% as shown in Figure 7a; however, for evaluating the response and recovery time the range of 35% to 85% was used as shown in Figure 9a.

Stability is another key feature to evaluate the performance of a sensor; therefore, we measured the stability of our fabricated humidity sensor by placing it inside jars with different levels of RH% at an applied voltage of 3V and observed the response of resulting current continuously for 1 h as shown in Figure 9b. The fabricated sensor was exposed to five different humidity levels including 15%, 46%, 60%, 72%, and 86% RH% inside their respective jars and the current was continuously measured. Figure 9b shows that the value of current remained stable at each humidity level.

We tested our fabricated sensor for the application of human health monitoring by exposing its surface to human breathing so that it can sense the water vapors. Our proposed sensor showed a fast response time with a quick recovery time and had the capability of measuring rapid and small RH% changes. The sensor response over normal breathing was tested. Figure 9c depicted the change of current with respect to normal human breathing. As the air was exhaled from the mouth, it contained water vapors; due to these water vapors’, conductivity of active layer increased resulting in more current flowing through it. During inhaling, conductivity decreases, hence proving that the GrF/ZnO-based humidity sensor exhibited an excellent response to breathing. The current change was in the microampere range; therefore, this proposed sensor can possibly be a promising candidate for health monitoring applications. 

Graphene and its derivate composites with ZnO have been reported by many authors for humidity sensing applications. Table 2 compares the performance of such sensors with our proposed device. This comparison table clearly shows that this proposed sensor has fast response and recovery time and is highly sensitive to humidity compared to other ZnO/Graphene composites.

To improve the humidity sensing properties of ZnO, its composition with other 2D materials like MoS_2_ [31] and WS_2_ [32] etc., has also been reported. Table 3 shows the performance comparison of this GrF/ZnO composite-based humidity sensor to other 2D materials/ZnO composite-based humidity sensors. The performance parameters including response time, recovery time, and sensitivity of the proposed sensor make it a better choice for humidity sensing as compared to other materials.

Furthermore, graphene composites with different materials like semiconductors [40], 2D materials [35], polymers [40], metal oxides [32], and biopolymers [37], have been reported for humidity sensors. A detailed comparison between this demonstrated GrF/ZnO composite and various other graphene composite-based humidity sensors is given in Table 4. The sensing performance sets the reported sensor apart from other humidity sensors. 

## 4. Conclusions

This work demonstrated an easy, solution-processed, and cost-effective way to fabricate a humidity sensor based on the composite of graphene flower and ZnO. Characterizations by FESEM, EDS, and XRD were carried out to completely understand the properties of GrF/ZnO composite. It was observed that GrF/ZnO composite had a high surface area with a porous nanowire-like structure, making it a suitable candidate for humidity sensing. Multiple electrical characterizations of the prepared humidity sensors were performed at different RH% levels. The proposed humidity sensor showed an increasing current and a decreasing impedance response with respect to increase in RH% from 15% to 86%. The demonstrated sensor showed a fast response with a current change of 7.7 μA/RH%. Moreover, the sensor revealed a fast recovery time and offered good stability over a wide range of humidity. The proposed sensor showed a measurable current change in response to normal human breath, making it a compelling candidate for health monitoring applications. GrF/ZnO composite has excellent morphological, chemical, and electrical properties that make it a useful candidate for many applications. 

## Figures and Tables

**Table 1:** Relative humidity (RH)% achieved in the airtight jar by the supersaturated solution of different salts.

Salt Solution	LiCl	CH_3_COOK	CaCl_2_	K_2_CO_3_	NaBr	CuCl_2_	NaCl	KCl	K_2_SO_4_
**RH%**	15%	23%	32%	46%	60%	67%	72%	80%	86%

**Table 2:** A comparison table of proposed work and the other reported ZnO/Graphene humidity sensors.

Active Material	Response Time (s)	Recovery Time (s)	Average Sensitivity (X/%RH)	Range	Reference
**ZnO/GrF**	**0.4**	**4**	**7.7** **μA/%RH**	**15–86%**	**This Work**
ZnO nanowires (NWs)/graphene quantum dots (GQDs)	27	12	40.16 kHz/%RH	20–90%	[43]
ZnO/Graphene Foam	10	15	33.3 Ω/%RH	20–95%	[42]
ZnO/PVP-RGO	12	3	-	15–95%	[44]

**Table 3:** Comparison table of GrF/ZnO and other 2D materials/ZnO composite-based humidity sensors.

Active Material	Response Time (s)	Recovery Time (s)	Average Sensitivity (X/%RH)	Range	Reference
**ZnO/GrF**	**0.4**	**4**	**7.7** **μA/%RH**	**15–86%**	**This Work**
ZnO/MoS2	1	20	-	11–95%	[31]
ZnO/WS2	74.51	25.67	101.71 fF/% RH	18–85%	[32]

**Table 4:** General comparison of proposed work with different graphene composite-based humidity sensors.

Active Material	Response Time (s)	Recovery Time (s)	Average Sensitivity (X/%RH)	Range	Reference
**ZnO/GrF**	**0.4**	**4**	**7.7** **μA/%RH**	**15–86%**	**This Work**
**Graphene film**	**0.125**	**0.125**	**-**	**11–95%**	[51]
Graphene oxide film	0.3	0.3	-	30–80%	[52]
BP/Graphene	9	30	73 Ω/%RH	15–70%	[35]
SiO2/PVA/Graphene	24	14.4	2.429 kHz/%RH	55–90%	[40]
Chitosan/GQD	36	3	39.2 Hz/%RH	11–95%	[41]
SnO2/RGO	102	6	1604.89 pF/%RH	11–97%	[33]
Non-woven fabric/GO	8.90	11.76	-	42–90%	[38]

## Supplementary Materials

The following are available online at https://www.mdpi.com/2079-4991/11/1/242/s1, Figure S1, shows the microscopic and digital camera image of screen-printed interdigitated electrodes. Figure S2, illustrates the setup used for measuring sensors electrical response with respect to RH%. Figure S3, shows an optimized FESEM and EDS analysis of ZnO thin film grown by the sol–gel method. Figure S4, shows the capacitance response of the GrF/ZnO-based humidity sensor at different frequencies. Impedance response of GrF/ZnO-based humidity sensor at different frequencies is depicted in Figure S5.
