# Supplementary material for: High-Performance Humidity Sensor Based on the Graphene Flower/Zinc Oxide Composite
Source: Nanomaterials (Basel). 2021 Jan 18;11(1):242. doi: 10.3390/nano11010242 (PMC7831307; doi:10.3390/nano11010242)
Supplement: Supplementary file 1 [file nanomaterials-11-00242-s001.pdf]

nanomaterials

MDPI

Supplementary Data

High-Performance Humidity Sensor Based on the Graphene Flower/Zinc Oxide Composite

Muhammad Saqib [1,*] Shenawar Ali Khan [1], Hafiz Mohammad Mutee Ur Rehman [1], Yunsook Yang [1], Seongwan Kim [1], Muhammad Muqeet Rehman [1] and Woo Young Kim [1,2,*]

Figure 1. is showing the microscopic image of screen-printed interdigitated electrodes that confirms the finger width of 40µm and distance between two fingers is 60µm. On right side printed IDE's camera picture is depicted.

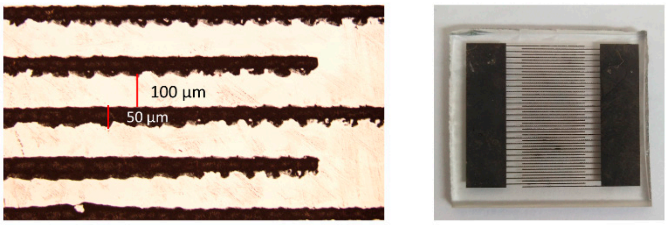

Figure S1. Microscopic and camera image of screen-printed interdigitated electrodes.

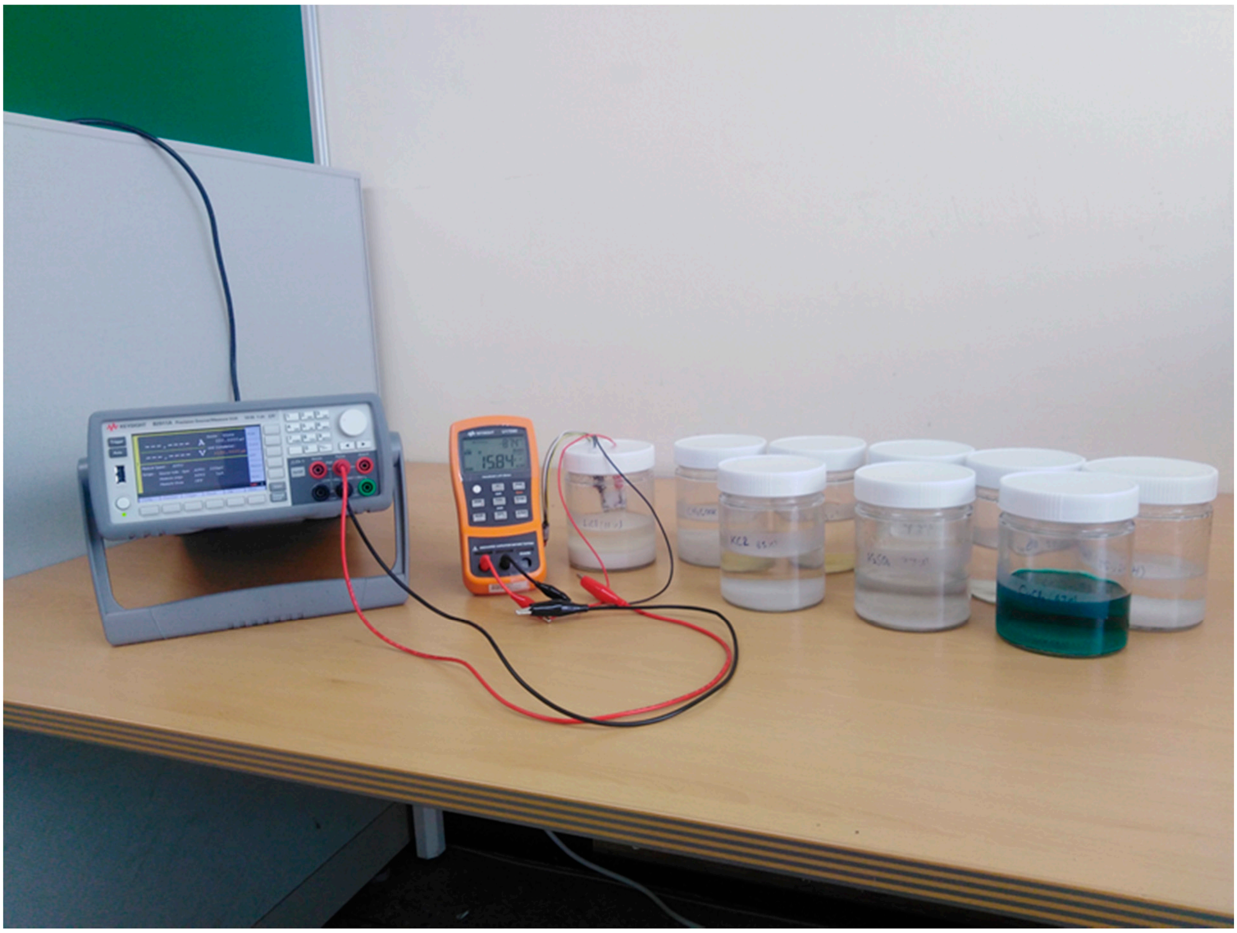

Figure S2. Setup used for measuring electrical response w.r.t RH%.

FESEM and EDS Analysis of Different Number of Spin-coated ZnO Thin Films

Figure S3, is showing an optimized analysis of ZnO thin film grown by sol-gel method, it is illustrating the effect of different number of spin-coatings on the morphological and elemental properties of ZnO thin films. The uniform ZnO thin films can be achieved by multiple number of spin-coatings, many authors reported different number of spin-coatings [1–3] to get uniform ZnO thin film. In this work, optimized study has been done, and we found that minimum seven times spin-coating is required to get a smooth ZnO thin film.

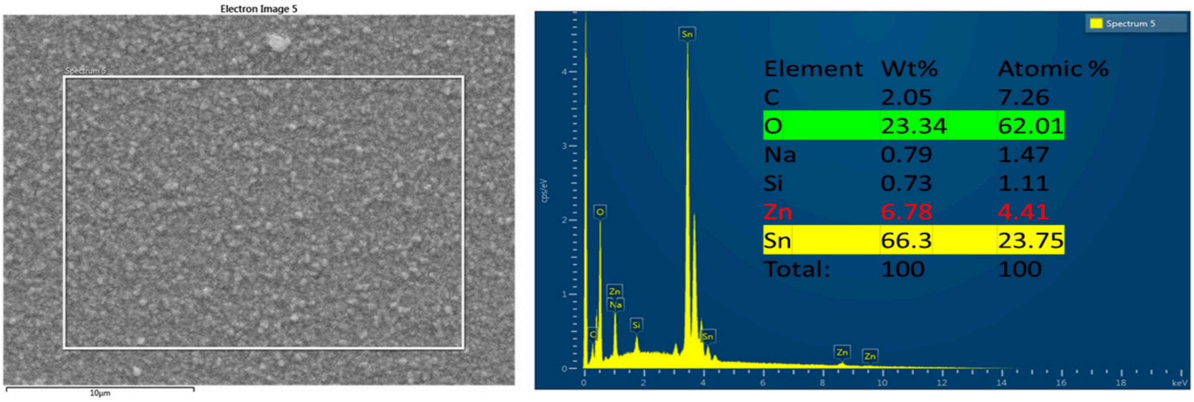

FESEM and EDS analysis of one time spin-coated ZnO thin film

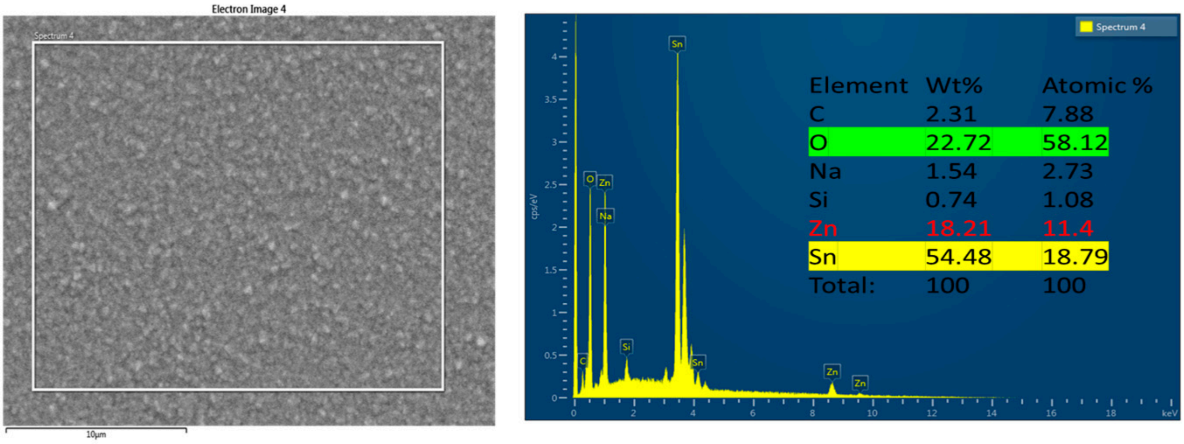

FESEM and EDS analysis of three time spin-coated ZnO thin film

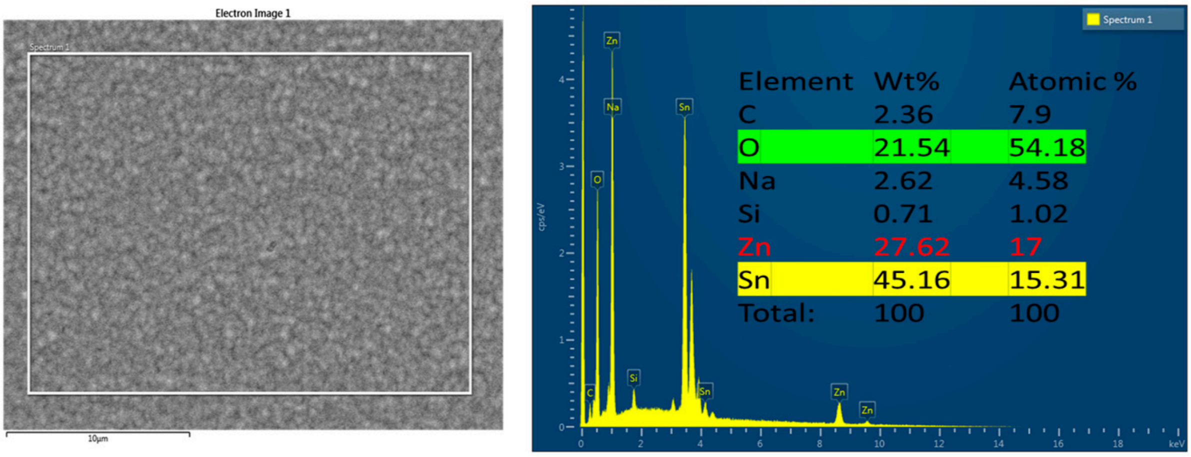

FESEM and EDS analysis of five time spin-coated ZnO thin film

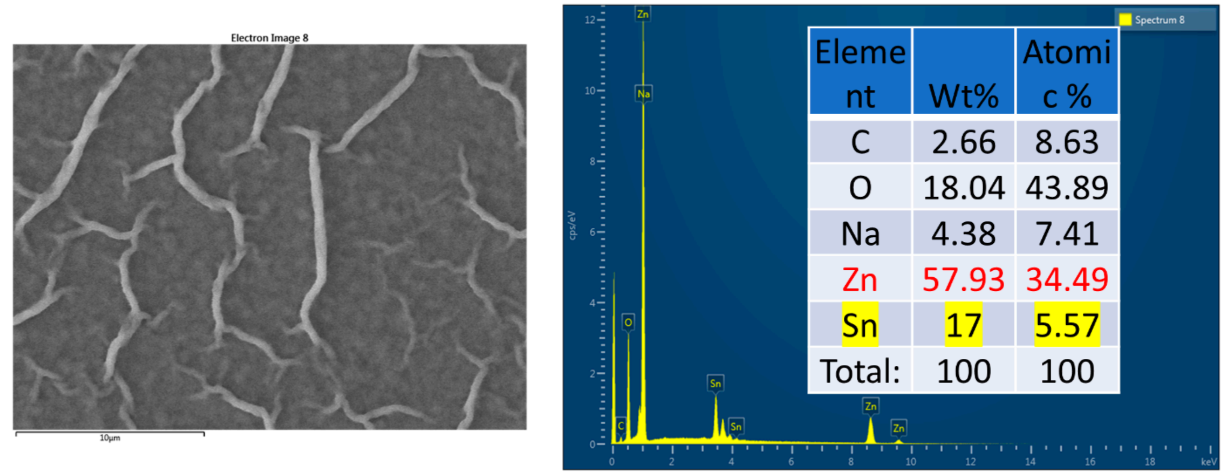

FESEM and EDS analysis of 7 times spin-coated ZnO thin film.

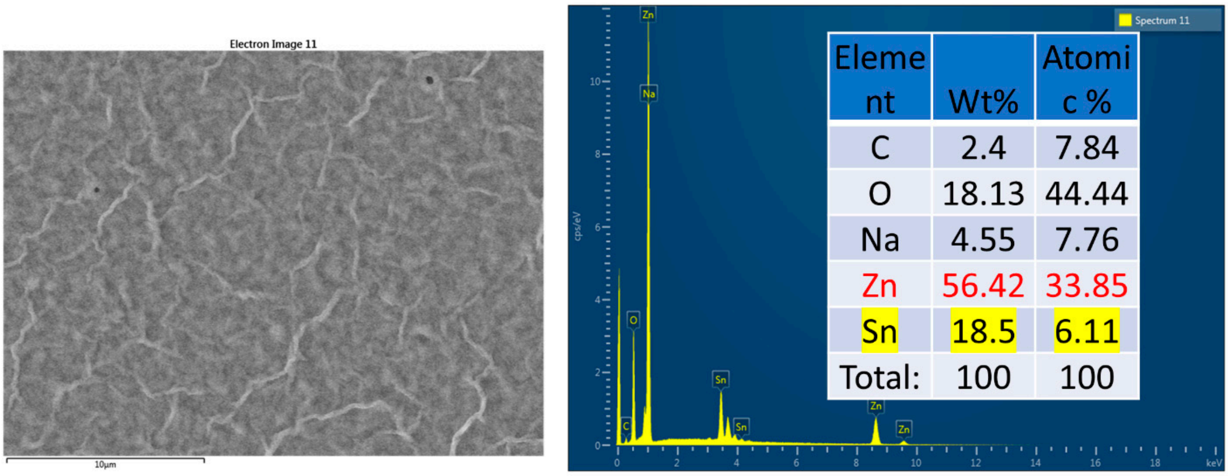

FESEM and EDS analysis of 9 times spin-coated ZnO thin film.

Figure S3. A detailed FESEM and EDS analysis of ZnO thin film.

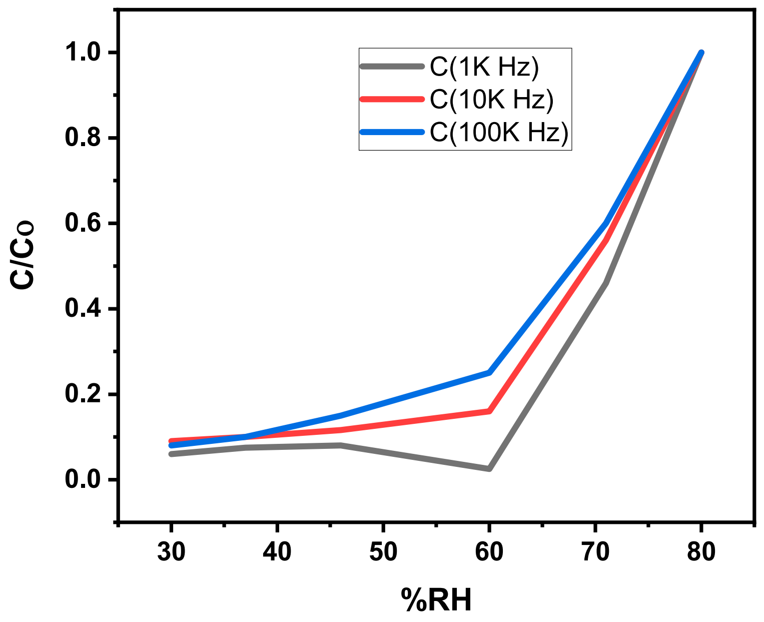

Figure S4. Normalized capacitance response of GrF/ZnO based humidity sensor at different frequencies.

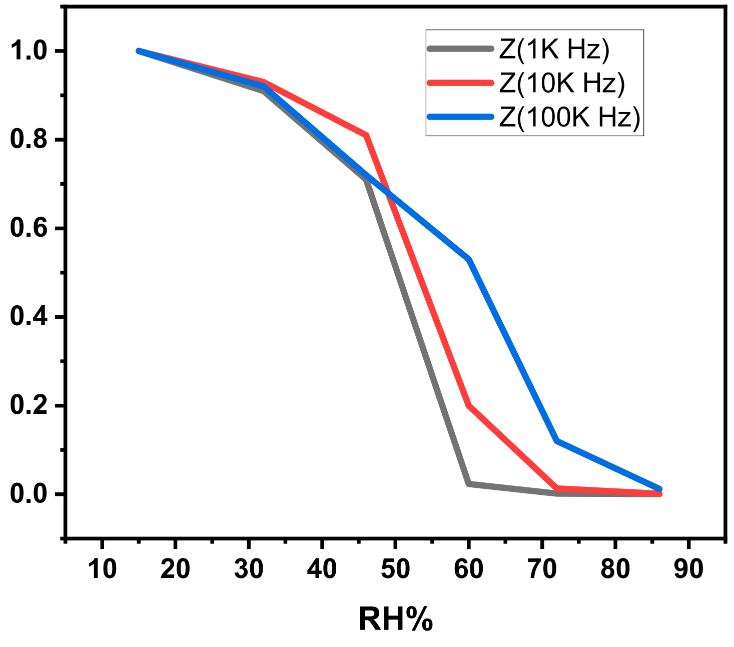

Figure S5. Normalized impedance response of GrF/ZnO based humidity sensor at different frequencies.

References

[1] A. J. Hashim, M. S. Jaafar, A. J. Ghazai, and N. M. Ahmed, "Fabrication and characterization of ZnO thin film using sol-gel method," *Optik (Stuttg).*, vol. 124, no. 6, pp. 491–492, Mar. 2013, doi: 10.1016/j.ijleo.2011.12.059.

[2] M. Z. Toe *et al.*, "Effect of ZnO seed layer on the growth of ZnO nanorods on silicon substrate," in

Materials Today: Proceedings, Jan. 2019, vol. 17, pp. 553–559, doi: 10.1016/j.matpr.2019.06.334.

[3] T. Amakali, L. S. Daniel, V. Uahengo, N. Y. Dzade, and N. H. de Leeuw, "Structural and Optical Properties of ZnO Thin Films Prepared by Molecular Precursor and Sol–Gel Methods," *Crystals*, vol. 10, no. 2, p. 132, Feb. 2020, doi: 10.3390/cryst10020132.
